# Three-dimensional imaging and computational quantitation as a novel approach to assess nerve fibers, enteric glial cells, mast cells, and the proximity of mast cells to the nerve fibers in human sigmoid mucosal biopsies from healthy subjects

**DOI:** 10.1016/j.jneumeth.2025.110436

**Published:** 2025-04-01

**Authors:** Pu-Qing Yuan, Tao Li, Swapna Mahurkar-Joshi, Jessica Sohn, Lin Chang, Yvette Taché

**Affiliations:** a Digestive Diseases Research Center, Vatche and Tamar Manoukian Digestive Diseases Division, David Geffen School of Medicine, UCLA, USA; b G Oppenheimer Center for Neurobiology of Stress and Resilience, Vatche and Tamar Manoukian Digestive Diseases Division, David Geffen School of Medicine, UCLA, USA; c VA Greater Los Angeles Healthcare System, Los Angeles, CA, USA

**Keywords:** Computerized quantitation, 3D imaging, Mast cells, Nerve fibers, Sigmoid mucosa biopsy, Tissue clearing

## Abstract

**Background:**

The visualization and quantitation of nerve fibers (NFs), enteric glial cells (EGCs), mast cells (MCs), and their spatial configurations in the human colonic mucosa represent considerable challenges due to the meshed network of these components and the arborizing of NFs in a three-dimensional (3D) structure.

**New method:**

We developed a novel approach combining tissue clearing, 3D imaging and computerized quantitation of NFs, EGCs and MCs in sigmoid mucosal biopsies of healthy subjects using a modified CLARITY tissue clearing protocol and adapting Imaris Surfaces Rendering Technology.

**Results:**

The cleared colonic biopsies are compatible with immunostaining using 10 marker antibodies and capable of generating 3D images rendering clear spatial views and computational quantitation of NFs, MCs, EGCs, in particular the proximity of MCs to NFs with Imaris 9.7–9.9.

**Comparison with existing methods:**

Our modified tissue clearing protocol shortened the membrane lipid removal time to 1 day from the original 1–2 weeks and total tissue clearing time to 3–4 days from the original 2–4 weeks. The 3D images displayed a clear spatial landscape of NFs, MCs and EGCs in the biopsies which cannot be portrayed with 2D images acquired from sections. Computerized quantitation is faster than measuring manually, allowing us to quantify a larger number of samples with less bias.

**Conclusion:**

The novel approach enables faster tissue clearing/immunolabeling, high-quality 3D imaging and precise computational quantitation of NFs, cells and proximity of MCs to NFs in human sigmoid biopsies which may allow new insight to detect alterations in colonic-related diseases.

## 1. Introduction

Recurrent abdominal pain is one of the main reasons for patients with gastrointestinal (GI) disorders, such as irritable bowel syndrome (IBS) and inflammatory bowel disease (IBD), to seek healthcare (Drossman, 2016; Spiegel et al., 2009; Knowles and Aziz, 2009). However, the treatment of abdominal pain is still an unmet need in part due to the incomplete knowledge of its underlying pathophysiology (Ford et al., 2024). The neural networks that mediate visceral pain in the human colon include the extrinsic efferent/afferent and intrinsic enteric nerve fibers (NFs) (Sengupta, 2009; Najjar et al., 2020). Additionally, mast cells (MCs) in the colonic mucosa are often found in close proximity to NFs and their crosstalk is thought to contribute to the visceral pain in patients with IBS (Barbara et al., 2004, 2007; Li et al., 2022; Barreau et al., 2008). Furthermore, recent evidence suggests that enteric glial cells (EGCs) adjacent to NFs are also involved in visceral hypersensitivity and chronic pain (Morales-Soto et al., 2023; Morales-Soto and Gulbransen, 2019). In the colonic mucosa, the NFs, EGCs and MCs form a three-dimensional (3D) complex anatomical and neurobiological network. A detailed visualization of the architecture and topography of this network in its spatial environment can help to detect their alterations in chronic GI diseases. However, 3D imaging of NFs and their spatial configurations with various cell populations, particularly MCs in the colonic mucosa, is challenging. This is due to the opacity of colonic mucosal tissue and related light scattering that restricts large volume imaging with microscopic resolution. The approach currently used for 3D imaging of the mucosal innervation relies largely on serial tissue sections, staining with histological markers or immunohistochemistry, and visualization via bright field or fluorescent microscopy (Laurino et al., 2023). While this approach has been the gold standard for over a century, it has significant shortcomings such as tissue deformation due to sectioning and a considerable amount of computational effort for 3D reconstruction, which are labor-intensive and time-consuming (Liu et al., 2021). Moreover, the long NFs that arborize in tortuous paths and different directions and are interwoven with each other in a 3D matrix together with other cell populations, cannot be easily portrayed with two-dimensional (2D) images acquired from tissue sections (4–6 μm each). The 2D images are also difficult to be precisely converted into 3D structures (Fu et al., 2013; Graham et al., 2020). Thus, the NFs, EGCs, MCs, and proximity of MCs to NFs in the colonic mucosa have not yet been thoroughly displayed and precisely quantitated.

To alleviate the shortcomings linked with the 2D visualization of nerve innervation, the approach of CLARITY (Clear Lipid-exchanged Acrylamide-hybridized Rigid Imaging/Immunohistochemistry/ In situ-hybridization-compatible Tissue-hYdrogel) has been developed which allows intact whole-tissue samples to be labeled and imaged without the need for serial sectioning (Chung and Deisseroth, 2013; Yang et al., 2014). This technique preserves spatial relationships and tissue microstructures by forming a tissue-hydrogel hybrid with increased permeability for deep optical interrogation. It is useful for 3D mapping and enables the immunofluorescence and maintenance of fluorophores during the imaging of large volumes for 3D reconstruction (Neckel et al., 2016). This method was initially developed for mapping the brain’s neuronal circuitry (Chung and Deisseroth, 2013; Yang et al., 2014; Neckel et al., 2016). Recently, we have adopted this approach and successfully developed a CLARITY/Immunofluorescence protocol applicable to the full thickness of human sigmoid colon samples dissected from postoperative specimens (Yuan et al., 2021). The 3D images and videos generated with this protocol demonstrated layer by layer the intrinsic cholinergic innervation in the human sigmoid colon including the mucosa in 3D microstructures. However, the colonic tissue specimens from patients with chronic GI disorders, such as IBS, are limited to colonic mucosal biopsies collected with sigmoidoscopy or colonoscopy, which are common endoscopic approaches for pathological diagnoses (Zuber, 2001). Roughly half of IBS patients undergo sigmoidoscopy (Talley et al., 1995) and a quarter of all colonoscopies performed in the United States are for IBS-related symptoms (Chey et al., 2010). Despite such a broad use of sigmoidoscopy/colonoscopy in the acquisition of mucosal biopsies to identify abnormalities in IBS patients, a tissue-clearing protocol specifically applicable for colonic mucosa biopsies and compatible with immunostaining using multiple marker antibodies to label NFs, EGCs and MCs is still lacking.

The traditional methods to count NFs, EGCs and MCs are performed on 2D images acquired from tissue sections manually via grid-based stereology and histomorphometry (Hunter et al., 2020). Though based on theoretically unbiased principles, these approaches largely depend on human data collectors, are labor intensive and prone to counting errors due to variable user training, subjectivity and work-related fatigue (West, 2012; Mouton, 2011). Moreover, as a variety of NFs enter into the colonic mucosa from the base and are distributed unevenly on the mucosa with their own distinct patterns, it would be a concern how a 2D image generated from a mucosal section crossing the different depths with a suboptimal orientation would represent them.

Therefore, to alleviate these deficiencies, we developed a new protocol applicable for clearing human sigmoid mucosal biopsies and compatible with immunostaining using marker antibodies for NFs from extrinsic sympathetic, parasympathetic, and sensory neurons and intrinsic enteric nervous system (ENS), as well as EGCs and MCs. 3D images and videos were generated using biopsy samples, with most of them displaying full-thickness mucosa vertically. A computerized approach was established for digital segmentation and computational quantification of NFs, MC, and EGC density as well as the shortest distances of each MC to NFs in 3D images of the whole biopsy tissue samples. This was achieved by applying Imaris 9.7–9.9 for Neuroscientists, a software for 3D/4D visualization, segmentation, and automated tracking of relevant components.

## 2. Methods

### 2.1. Subjects

Eight healthy subjects (four men and four women, aged 18–55 years) were recruited primarily by community advertisement. They had no personal or family history of IBS or other chronic abdominal or somatic pain conditions. Additional exclusion criteria for all subjects included infectious or inflammatory disorders, active psychiatric illness over the past six months assessed by the Mini-International Neuropsychiatric Interview (M.I.N.I.) for the Diagnostic and Statistical Manual of Mental Disorders-IV (DSM-IV) (Sheehan et al., 1998), use of laxatives, antidiarrheals, narcotics, antidepressants, or corticosteroids in the past six months, or current tobacco or alcohol abuse. Participants were compensated. This study was approved by the UCLA Institutional Review Board (20–000241), and all subjects signed a written informed consent form prior to starting the study.

### 2.2. Sigmoidoscopy

All participants underwent a flexible sigmoidoscopy procedure with sigmoid mucosal biopsies collected at 30 cm from the anal verge. Tap water enemas were used as bowel preparation. Participants were instructed to hold aspirin and nonsteroidal anti-inflammatory drugs for 72 h before the procedure. Each mucosa biopsy sample had a volume of ~ 6×3×1–2 mm (length × wide × thickness) and was processed for the CLARITY tissue clearing procedure. The interval time between the biopsy collection and the beginning of CLARITY was about 60–90 min.

### 2.3. Tissue clearing of sigmoid mucosal biopsies

The protocol of passive CLARITY that we developed previously for full thickness of human sigmoid colon (Yuan et al., 2021) was modified to clear the whole sigmoid mucosal biopsies. The details of this protocol have been shared in protocol io (Yuan et al., 2024). Briefly, the biopsy samples were flattened with the mucosa facing up on the cardboard and immersed in 4 % paraformaldehyde [Sigma-Aldrich, St Louis, MO; Catalog number (Cat No.) P1648–1KG] in phosphate-buffered saline (PBS) at 4°C overnight. On the second day, the samples were immersed in an ice-cold hydrogel solution known as A4P0 (Yang et al., 2014), made from a mixture of 4 % acrylamide (BIO-RAD, Hercules, CA; Cat No. 1610140) and 0.25 % VA044 (Wako, Richmond, VA; Cat No. 27776–21–2) in 1x PBS (pH 7.4). The samples were then maintained at 4°C for one day to facilitate the hydrogel-tissue hybridization. Each sample was transferred to a 15 ml conical tube containing 5 ml of new hydrogel solution to replace all oxygen (known to inhibit acrylamide polymerization) with pure nitrogen gas. The hydrogel polymerization was initiated by submerging the conical tube containing the tissue samples in a water bath at 37°C (Precision Scientific Water Bath Model 182, Thermo Scientific, Marietta, Ohio) for 3 h. The excess hydrogel monomers inside the samples were washed out on a shaker/rotator plate (70 rpm, New Brunswick Scientific Co., Edison, NJ) with a clearing solution containing 4 % sodium dodecyl sulfate (SDS, Sigma-Aldrich; Cat No. L3771–1KG) in sodium borate buffer (200 mM, pH 8.5) (Sigma-Aldrich; Cat No. B7901–1KG) at 37°C for about 1 day until clearing is achieved. Thereafter, samples were placed in solution containing 1 % SDS in sodium borate buffer at 37°C for 3 h and then in 0.1 % Triton-X 100 (Sigma-Aldrich; Cat No. T8787–ml) in 1x PBS (TPBS, pH 7.4) on a shaker plate (New Brunswick Scientific Co.) for 1 h (37 °C, 70 rpm) to wash out sodium dodecyl sulfate micelles. The cleared samples were immersed overnight at 4°C in 20 % sucrose in PBS for cryoprotection and subsequently embedded in optimal cutting temperature compound (OCT, Thermo Fisher Scientific, Waltham, MA, Cat No. 23–730–571) on dry ice. The OCT blocks with the cleared samples were cut in vertical sections (200 μm) using a cryostat (Leica CM3050 S, Leica Microsystems Nussloch GmbH, Nussloch, Germany) at −20°C.

### 2.4. Immunohistochemistry

The immunohistochemistry of CLARITY-cleared sigmoid mucosal samples was adapted from the protocol previously described (Yuan et al., 2021). Sections were transferred into Corning^™^ Costar^™^ Flat Bottom 24 Well Plates (Fisher Scientific, Hampton, NH) for free-floating immunohistochemistry with sections floating in solution for antibody incubation and washing under gentle shaking (90 rpm, Corning^™^ LSE^™^ Low Speed Orbital Shaker, Fisher Scientific, Canoga Park, CA). The marker antibodies include protein gene product 9.5 (PGP9.5) for pan-nerve fibers, substance P (SP) mainly for extrinsic primary afferent fibers, vasoactive intestinal peptide (VIP) for intrinsic secretomotor nerve fibers, vesicular acetylcholine transporter (VAChT) for cholinergic nerve fibers with extrinsic and intrinsic origin, human peripheral choline acetyltransferase (hpChAT) for intrinsic cholinergic nerve fibers, calbindin (Calb) for intrinsic primary afferent nerve fibers, tyrosine hydroxylase (TH) and neuropeptide (NPY) for sympathetic nerve fibers, S100β for EGCs and tryptase for mucosal MCs (Table 1). These primary antibodies have been well characterized and validated for immunohistochemistry with human colonic samples prepared with different methods including CLARITY tissue clearing technique by our group and others (Brookes et al., 2022). After immersion in 10 % normal donkey serum in 0.3 % Triton-X 100 PBS at room temperature (RT) for one day to block non-specific interactions and to enhance permeabilization, single immunolabeling of TH, VIP, NPY, S100β or double immunolabeling of PGP9.5, SP or Calb with tryptase, and VAChT with hpChAT were conducted by incubation with primary antibodies (Table 1) at 4°C for 5 days. After washing the samples with PBS on a shaker at RT for one day, they were incubated with fluorochrome-labeled secondary antibodies (Table 1) while continuously shaking at RT for another day. Following this incubation, the samples were washed with PBS again at RT for an additional day. Negative control samples were subjected to the same procedures without primary antibody. Samples were then immersed in a custom-made refractive index matching solution (RIMS, refractive index 1.46) containing 88 % Histodenz (Sigma-Aldrich; Cat No. D2158–100G) in 0.02 M phosphate buffer with 0.01 % sodium azide (Sigma-Aldrich; Cat No. 438456, pH 7.5) at 4°C (Yang et al., 2014) for 1 day. Sections were mounted with fresh RIMS in a sealed watertight well prepared with iSpacers (SunJin Lab, Hsinchu City, Taiwan) which are made from different thickness adhesive tape.

### 2.5. 3D Imaging and quantification

Z-stack images with a 708 × 708 μm frame (20 ×objective) were acquired from CLARITY-cleared samples with Zeiss LSM 710 confocal microscopes (Carl Zeiss Microscopy, LLC, White Plains, NY). The Alexa Fluor^®^ 488 and 555 were excited using a 488 nm Argon laser and a 561–10 nm diode-pumped solid-state laser, respectively. To allow the double immunolabeling to be interpreted by color-blind readers, the red color in dual-labeled images was converted to the magenta color. The Z-axis intervals were 1 μm with depths 150–200 μm. The confocal parameters were determined, and the same setting was applied for each sample. To minimize fading and photobleaching, we first identified the region of interest and optimized the focus using transmitted light before fluorescence imaging. We then employed bi-directional scanning, a feature of the Zeiss LSM 710 confocal microscope, which enabled simultaneous excitation of the Alexa Fluor^®^ 488 and 555 dyes from two scanning directions and achieved a scanning rate of approximately 8 frames per second at a resolution of 512 × 512 pixels. These measures doubled the scanning speed, significantly reduced light exposure and excitation laser intensity, and effectively minimized photobleaching. The Z-stack images were collected using Zen image collection software (Carl Zeiss Microscopy) and processed for 3D reconstruction using Imaris 9.7–9.9 for Neuroscientists (Bitplane Inc., Concord, MA).

Each 3D image used for quantitation showed the full thickness of the mucosa with vertical orientation from basal to the top. The 5–6 3D images of each marker-labeled NFs, EGCs, and MCs generated from each subject were quantitated. A computerized approach for measurement of the densities of NFs, EGCs, MCs, and the proximity of MCs to NFs in 3D images of the sigmoid mucosal biopsies was developed by adapting Imaris 9.7 Surfaces Rendering Technology (To access the content, please copy and paste the following URL in a browser: https://imaris.oxinst.com/products/imaris-for-neuroscientists). A step-by-step computational workflow has been detailed and shared in protocol io (Li et al., 2024). Briefly, the full-thickness mucosa in 3D image was contoured and separated from the submucosa with ImarisContour function. The surfaces of nerve fibers labeled by marker antibodies were created automatically with the ImarisSurface function. The volumes of contoured mucosa and the surface-masked nerve fibers were automatically measured using the software Imaris 9.7–9.9. The density of each subclassified nerve fiber was calculated and expressed as a percentage of its volume in the mucosa volume (v/v, %). The density of EGCs was measured with the same protocol as described above and expressed as a percentage of EGC volume in the mucosa volume (v/v, %). The mast cells were spotted with ImarisSpot function and automatically counted with Imaris 9.7–9.9. The density of MCs was calculated and expressed as the number of spots per volume of mucosa (MCs/μm^3^). The shortest distances of the centers of each individual spot (MCs) to the surface-marked PGP9.5-, SP- and Calb-immunoreactive (ir) NFs in 3D images were automatically measured and plotted correspondently with Imaris 9.7–9.9. The shortest distance with values equal or less than 5.2 μm was defined as the contact to NFs. This is based on the sizes of human colonic mucosal MCs measured in this study with an average diameter 10.4 μm. The proximity of MCs to the NFs was expressed as a percentage of MCs with contact to NFs in the total MCs (%).

### 2.6. Statistical analysis

All data are expressed as mean ± SEM. Statistical analysis of the difference between two groups was performed by two-tailed student’s *t*-test. Multiple group comparisons were performed with one-way analysis of variance (ANOVA) followed by Tukey’s post hoc test. A p value < 0.05 was considered statistically significant.

## 3. Results

### 3.1. Tissue clearing of human sigmoid mucosal biopsies

**Fig. 1.**
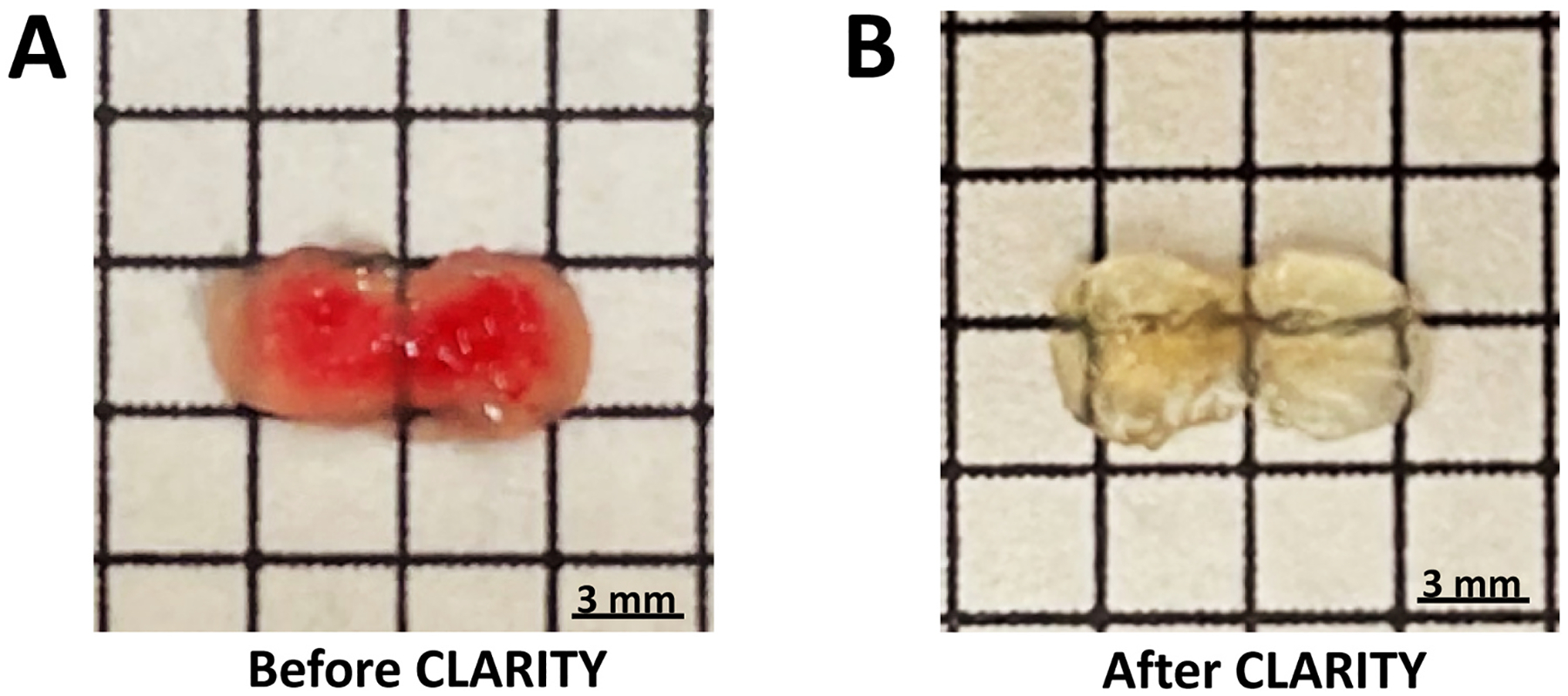
Human sigmoid mucosal biopsy sample before (A) and after (B) clearing with the modified passive CLARITY protocol. The full-thickness human sigmoid mucosa biopsy specimen collected from a healthy man was cleared within 4 days making the grid lines visible through the completely transparent biopsy sample (Fig. B). This allowed us to image deeply and obtain high-quality Z-stack confocal images for 3D reconstruction.

**Fig. 2.**
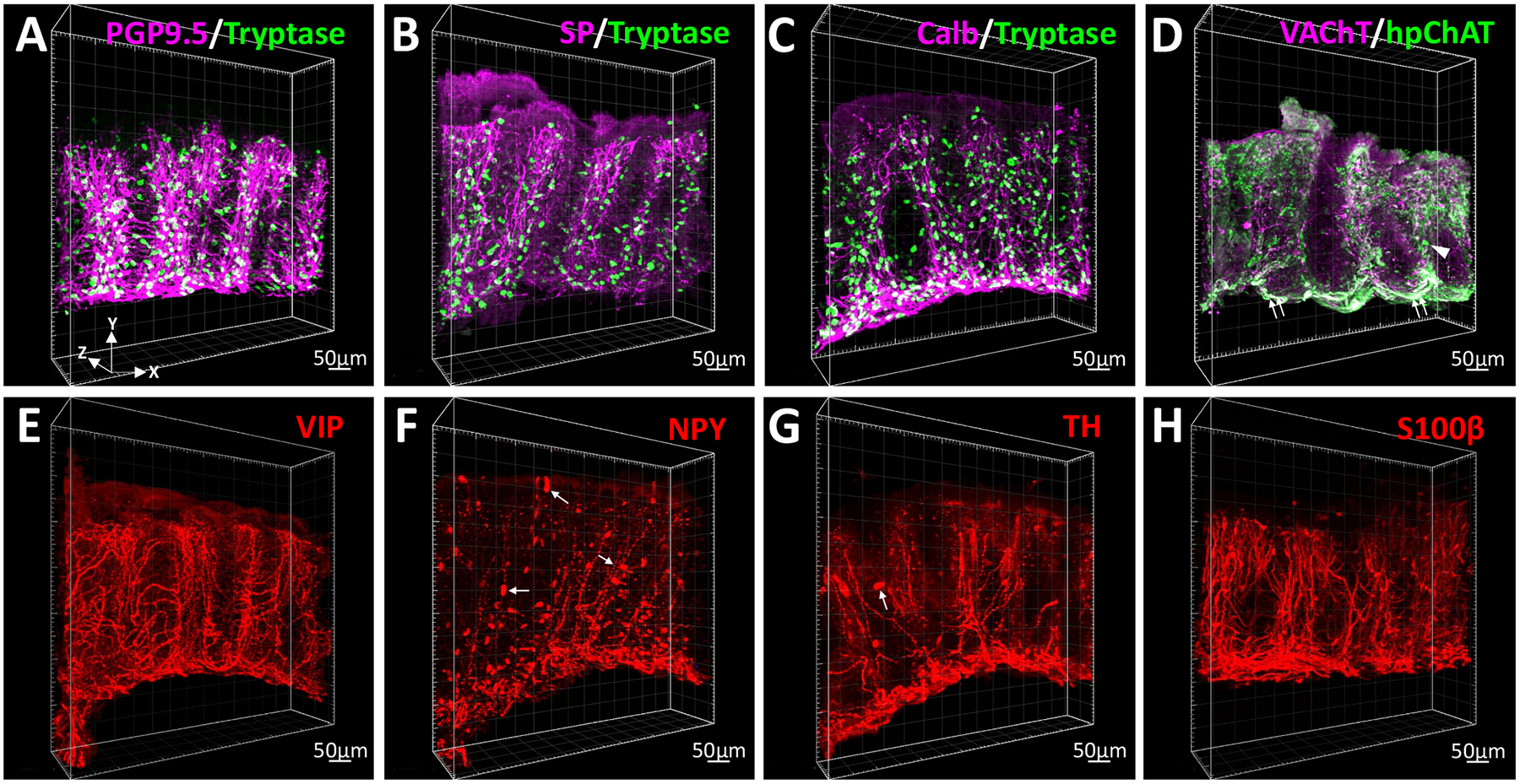
3D images illustrating nerve fibers (NFs), enteric glial cells (EGCs), mast cells (MCs), and the spatial configurations of NFs and MCs with XYZ axis visualizations of the 3D volumes in the sigmoid mucosa biopsies collected from a healthy man. Each 3D image was generated from 150 to 200 optical sections (Z-stack) with 1 μm apart (20 × objective) and 708 × 708 μm frame acquired with LSM710 confocal microscope. The NFs, EGCs and MCs were immunohistochemically labeled with marker antibodies including protein gene product 9.5 (PGP9.5) for pan-NFs, and tryptase for MCs, substance P (SP) mainly for extrinsic primary afferent NFs, calbindin (CalB) for intrinsic primary afferent NFs, vesicular acetylcholine transporter (VAChT) for cholinergic NFs with extrinsic and intrinsic origin, human peripheral choline acetyltransferase (hpChAT) for intrinsic cholinergic NFs, vasoactive intestinal peptide (VIP) for intrinsic secretomotor NFs, neuropeptide (NPY) and tyrosine hydroxylase (TH) for extrinsic sympathetic NFs, S100β for enteric glial cells (EGCs) and tryptase for colonic mucosalMCs. A-C: double labeling of PGP9.5, SP, or Calb (magenta color) with tryptase (green color). D: double labeling of VAChT (mgenta color) with hpChAT (green color). Double arrows indicate a few hpChAT-ir fibers co-labeled with VAChT, while an arrowhead points to a hpChAT-ir cell body” after “with hpChAT (green color). E-H: single labeling of VIP, NPY, TH and S100β (red color). The arrows point to the endocrine cells labeled with NPY and TH after (red color). Bar scale: 50 μm.

Using the CLARITY tissue clearing protocol developed in this study, the sigmoid mucosa biopsy specimens of healthy subjects were cleared within 3–4 days making the grid lines visible through the completely transparent biopsy sample (Fig. 1A, B). The vertical sections (200-μm) generated from cleared biopsy samples enabled us to visualize the colonic innervation and distributions of EGCs and MCs from the basal to the top within the full thickness of colonic mucosa (Fig. 2A–H).

### 3.2. 3D imaging of nerve fibers (NFs), enteric glial cells (EGCs) and mast cells

The 3D images and videos generated from 150 to 200 optical sections with 1 μm interval per sample showed clear spatial views of innervation with NFs, distributions of MCs and EGCs labeled with marker antibodies, and the configuration of MCs with NFs in the sigmoid mucosa biopsy samples (Fig. 2A–H, Suppl. Videos 1–6). The NFs labeled with the pan-neuronal marker PGP9.5 form a dense micro-network in the mucosal lamina propria (Fig. 2 A, Suppl. Video 1). Most SP-labeled NFs were orientated parallel to the course of the corresponding crypts and projected from the basal to the top of the mucosa reaching to the epithelia (Fig. 2B, Suppl. Video 2). The Calb-labeled NFs were denser at the lower third area of the mucosa (Fig. 2 C, Suppl. Video 3). The double labeling with a rabbit antibody against VAChT, a marker for cholinergic innervation with extrinsic and intrinsic origin and the mouse antibody against hpChAT, a marker specific for intrinsic cholinergic innervation, showed VAChT-labeled NFs with fine and varicose morphology projecting to the upper mucosa, while hpChAT-labeled NFs displayed intensive immunoreactivity that was mostly distributed in the lower area. A few hpChAT-ir fibers co-labeled with VAChT and hpChAT-ir cell bodies were also observed (Fig. 2D). VIP labeled NFs were dense and surround the crypts (Fig. 2E, Suppl. Video 4). NPY- and TH-labeled NFs showed a high density in the basal area of the mucosa (Fig. 2 F, 2 G). NPY and TH immunostaining was also seen in the endocrine cells located in the epithelia and crypts (Fig. 2 F, 2 G, Suppl. Video 5).

EGCs labeled by S100β showed small soma with star or spindle shapes and long branching processes in the mucosal lamina propria (Fig. 2H, Suppl. Video 6). Fig. 2A–C displayed the spatial configuration of MCs labelled by tryptase with NFs labeled with PGP9.5, SP or Calb. In 3D images, MCs were clearly observed, distributed ubiquitously in the mucosal lamina propria, unequally with a higher density in the lower two third areas and accumulating around NFs. There were rare MCs in the area without NFs (Fig. 2A–C). The videos with a 360-degree view showed that most MCs were close or in contact to PGP9.5-, SP- or Calb-ir NFs from all directions (Suppl. Videos 1–3).

### 3.3. Digital segmentation and computational quantitation

#### 3.3.1. The densities of nerve fibers (NFs), mast cells (MCs) and enteric glial cells (EGCs).

**Fig. 3.**
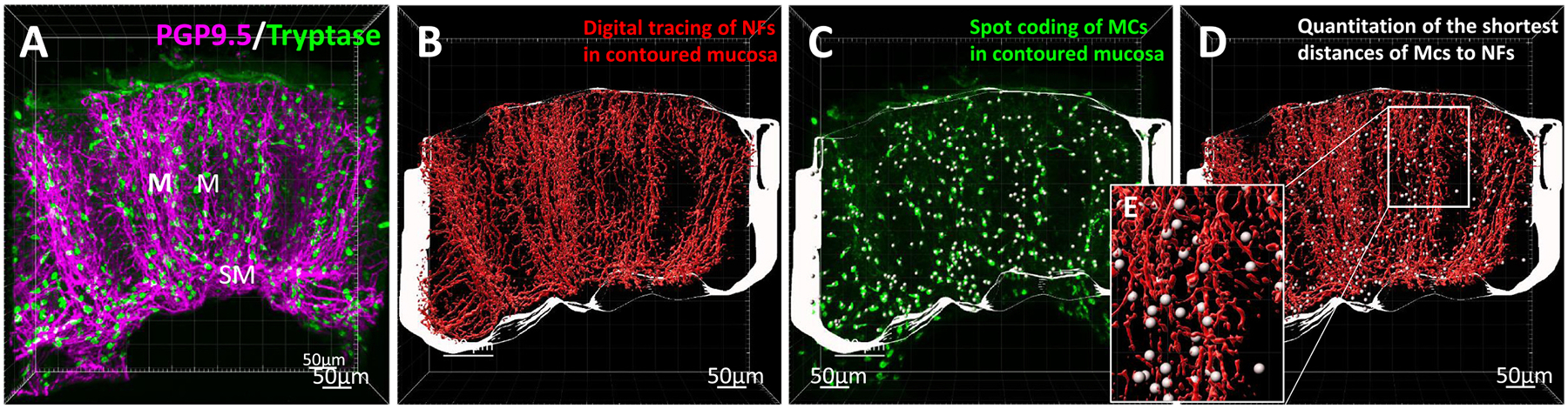
Segmentation and measurement of the nerve fibers (NFs), mast cells (MCs) and the proximity of MCs to NFs in 3D images of human sigmoid colonic mucosa biopsies using a computerized approach developed in this study. A: a 3D image was generated from 170 confocal optical sections (Z-stack) with 708 × 708 μm frame and 1 μm apart (20 × objective) showing NFs and MCs labeled with antibodies of pan-neuronal marker PGP9.5 (magenta color) and mucosal MCs marker tryptase (green). B, C: NFs and MCs were digitally segmented by using Imaris 9.7–9.9 surface and spot functions respectively. The mucosa was contoured with ImarisContour function. The volumes of contoured mucosa (μm^3^) and NFs (μm^3^), and the number (Nb) of the MCs were automatically measured and the densities of NFs and MCs were calculated and expressed as the percentage of the NF volume in the volume of contoured mucosa (v/v,%) and the Nb of MCs per unit volume of contoured mucosa (MCs/μm^3^). D. The segmented NFs and MCs shown in 3D image and the insert with high magnification (E) were used for the assessment of the proximity of MCs to NFs. The shortest distances of the centers of each individual spot (MCs) to the surfaces of NFs in 3D images were automatically measured and plotted correspondently. The shortest distance with values equal or less than 5.2 μm was defined as the contact to NFs. The proximity of MCs to the NFs was expressed as a percentage of MCs with contact to NFs in the total MCs (%). Bar scale: 50 μm.

**Fig. 4.**
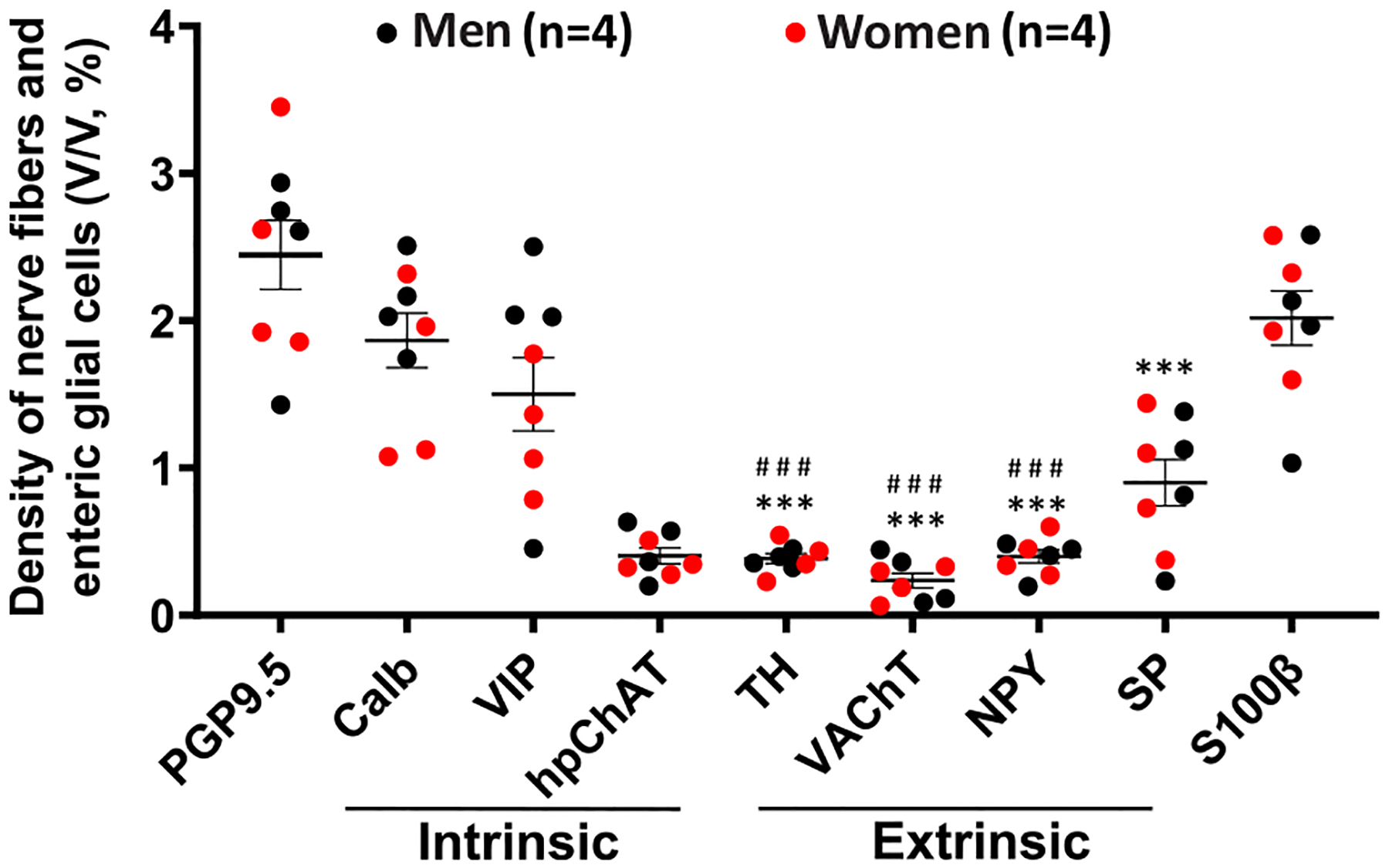
Quantitative analysis of densities of nerve fibers (NFs) and enteric glial cells (EGCs) in 3D images of human sigmoid colonic mucosa biopsies using the computerized approach developed in this study. The NFs were subclassified by immunolabeling with specific marker antibodies against as PGP9.5 (protein gene product 9.5) for pan-nerve fibers, Calb (calbindin) for intrinsic primary afferent nerve fibers, VIP (vasoactive intestinal peptide) for intrinsic secretomotor nerve fibers, hpChAT (human peripheral choline acetyltransferase) for intrinsic cholinergic nerve fibers, VAChT (vesicular acetylcholine transporter) for cholinergic nerve fibers with extrinsic and intrinsic origin, NPY (neuropeptide) and TH (hydroxylase) for extrinsic sympathetic nerve fibers, SP (substance P) for extrinsic primary afferent fibers and enteric glial cells (EGCs) were labeled with S100β. The densities of NFs and EGCs were calculated from 5 to 6 3D images labeled with each marker per subject and averaged from 8 adult health subjects including 4 of each sex. Data were scatter-plotted as mean ± SEM with individual value of each subject spotted and men and women marked as black and red respectively. Multiple group comparisons were performed with one-way analysis of variance (ANOVA) followed by Tukey’s post hoc test. A p value < 0.05 was considered statistically significant. * ** p < 0.001 vs. Calb;, ### p < 0.001 vs VIP. No significance was detected between men and women.

**Fig. 5.**
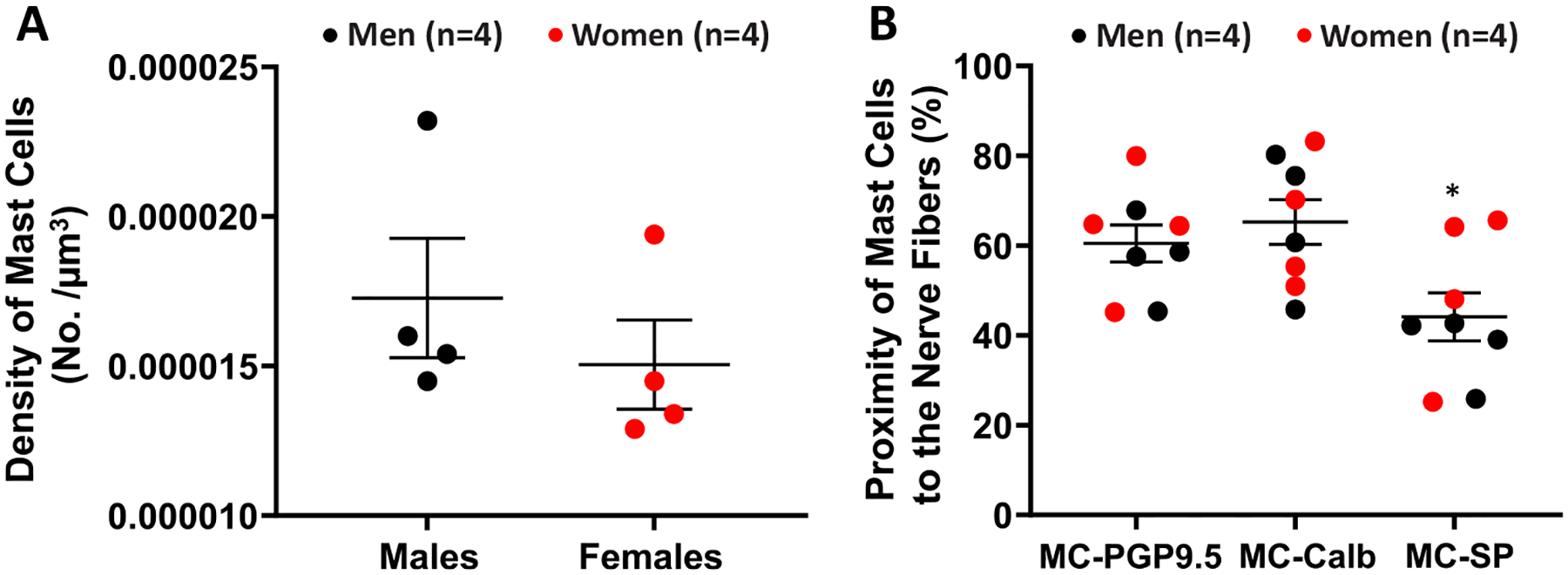
Quantitative analysis of mast cells (MCs) densities and proximity of MCs to nerve fibers (NFs) in 3D images of sigmoid colonic mucosa biopsies of healthy adult subjects using the computerized approach developed in this study. A: the density of MCs (MCs/μm^3^) and B: the proximity of MCs to protein gene product 9.5 (PGP9.5), calbindin (Calb) and substance P (SP)-labeled NFs (MC-PGP9.5, MC-Calb, MC-SP) expressed as percentage of MCs with contact to NFs in the total MCs (%). 5–6 of 3D images of each double immunolabeling per subject were calculated and averaged from 8 subjects with 4 of each sex scatter-plotted as mean ± SEM. Each spot represents an individual value of each subject with man and woman marked as black and red respectively. p < 0.05 vs. MC-Calb. No significance was detected between sex in both the density of MCs and the proximity of MCs to PGP9.5, Calb and SP-labeled NFs.

Using ImarisSurface and ImarisSpot Functions, the NFs and EGCs were digitally segmented by creating surfaces as red color over them (Fig. 3A, B) while the MCs were marked in spots as white color in the centers of each individual MCs in 3D images (Fig. 3A, C). The entire mucosa was outlined and separated from the submucosa in each 3D image (Fig. 3B). The volumes of surface-marked NFs, EGCs, outlined mucosa, and numbers of spotted MCs were measured automatically with Imaris 9.7–9.9. The labeling of total NFs by PGP9.5 and EGCs by S100β represent about 2.4 % and 2.0 % respectively of the total mucosa volume of the sigmoid mucosa in healthy subjects. The densities of intrinsic NFs labeled by Calb or VIP are significantly greater than those of extrinsic NFs labeled by TH and NPY or those of VAChT and SP both being mostly of extrinsic origin (Fig. 4). The hpChAT-labeled intrinsic NFs show less density than those of Calb or VIP-labeled intrinsic NFs while they were similar to that of TH or VAChT-labeled extrinsic NFs (Fig. 4, Table S1). The densities of NFs, EGCs and MCs did not show significant differences between men and women (Figs. 4, 5A, Table S1).

#### 3.3.1. The proximity of mast cells (MCs) to the nerve fibers (NFs)

The proximity of MCs to Calb-labeled NFs (MC-Calb) is shown in Fig. 2 C. When expressed as a percentage of MCs with contact to Calb-NFs in the total MCs (%), the MC-Calb proximity is greater than that of MCs to SP-labeled MFs (MC-SP) (p < 0.05) (Fig. 5B). There were no significant differences in the proximity of MCs to PGP9.5-labeled NFs (MC-PGP9.5), MC-Calb and MC-SP between men and women (Fig. 5B, Suppl. Table 1).

## 4. Discussion

This study introduced a novel method enabling fast tissue clearing/immunolabeling, high-quality 3D imaging and precise computational quantitation of NFs, EGCs, MCs and the proximity of MCs to the NFs in the mucosal biopsies of sigmoid colon in healthy subjects. This method was developed by modifying the CLARITY tissue clearing protocol that we established previously for the human colonic enteric nervous system (Yuan et al., 2021) and adapting Imaris Surfaces Rendering Technology for automated segmentation, tracking and quantitative analysis of targeted components with Imaris 9.7–9.9. Our new method shortened the tissue clearing time by 3–4 days from originally taking 2–4 weeks. The cleared tissue samples are compatible with immunostaining using 10 selected marker antibodies. 3D images and videos displayed a clear spatial landscape of NFs, MCs, EGCs and their configurations in the sigmoid colonic mucosa which cannot be achieved with 2D images acquired from sections. Computerized quantitation is much faster than measuring manually and allows us to quantitate a larger number of samples with less bias. A detailed protocol for tissue clearing/immunostaining/3D imaging and a step-by-step computational workflow of segmentation and quantitation have been shared in protocol io with DOIs (Yuan et al., 2024; Li et al., 2024). Links are provided at each relevant section.

### 4.1. Optimization of the CLARITY protocol for clearing sigmoid mucosal biopsies to be compatible with immunohistochemistry

As a novel tissue-clearing method, the original CLARITY has several technical limitations. The passive CLARITY is time-consuming as it relies on diffusion that requires several weeks to achieve clearing and labeling of large-scale specimens while the active CLARITY requires more equipment and supervision associated with the use of electrophoresis. The optimization of the CLARITY technique focuses on simplifying the procedures and shortening the processing time to suit different specimens (Yang et al., 2014; Kim et al., 2015; Lee et al., 2016; Zheng and Rinaman, 2016; Woo et al., 2016; Lai et al., 2017; Tian et al., 2021; Vigouroux et al., 2017; Ueda et al., 2020; Azaripour et al., 2016). In the present study, we utilized a CLARITY hydrogel recipe composed of a mixture of 4 % acrylamide and 0.25 % VA044 in 1x PBS, referred to as A4P0 (Yang et al., 2014), for hydrogel-tissue hybridization. This is based on evidence indicating that omitting paraformaldehyde and bis-acrylamide from the original hydrogel composition, which included 4 % paraformaldehyde, 4 % acrylamide, 0.05 % bis-acrylamide, and 0.25 % VA-044 (Chung and Deisseroth, 2013), could potentially increase the hydrogel mesh porosity and improve SDS transport speed (Yang et al., 2014). We adopted and optimized the clearing protocol, initially developed for surgically dissected full-thickness human sigmoid colon samples (Yuan et al., 2021), to small biopsy samples (6×3×1–2 mm dimensions). This adaptation reduced the hydrogel embedding time from 3 days to 1 day and shortened the membrane lipid removal with 6 % SDS from 2 to 4 weeks to 3 days, allowing for biopsy tissue clearing within approximately 4 days. However, we noted that some marker antibodies, including SP, VIP, VAChT, NPY, and tryptase, yielded dim or no immunostaining, despite these antibodies, along with all others selected in this study, being well characterized and validated for use in immunohistochemistry with human colonic samples prepared using various methods, including the CLARITY tissue clearing technique (Brookes et al., 2022). In light of this, we further reduced the SDS concentration in the clearing solution from 6 % [used for surgically dissected human sigmoid colon (Yuan et al., 2021)] to 4 %. This adjustment was made considering that SDS, a strong ionic detergent, can damage tissue integrity and inhibit antibody-antigen binding (Tian et al., 2021; Wang et al., 2022). The excess hydrogel monomers were washed out using 4 % SDS on a shaker/rotator plate (70 rpm) at 37°C for one day. Finally, samples were washed with 1 % SDS for 3 hours and then with 0.1 % Triton-X 100 in 1x PBS for 1 hour to remove SDS micelles, following the same conditions as with 4 % SDS. These optimization steps ensured that the integrity of the biopsy tissue was maximally preserved and underwent minimal transformation during the clearing process. This was particularly beneficial for maintaining antigenicity and improving immunostaining. As a result, the biopsy samples were not only optically transparent after four days, making them suitable for deep imaging, but the immunostaining using all 10 marker antibodies was also significantly enhanced. The 3D images and videos generated from these cleared biopsy samples provided sharp spatial views of NFs labeled with all marker antibodies including those with dim or without immunostaining before further optimization. This allowed us to visualize distinct axonal projections and distribution patterns. Additionally, the MCs were successfully labeled with tryptase antibody which failed using an unoptimized protocol. Of note, biopsy samples embedded with 4 % acrylamide and 0.25 % VA-044 exhibited slightly more swelling than those processed with the original hydrogel solution. However, they shrank back to their original size when immersed in the 0.1 % Triton-X 100 PBS as previously reported (Yang et al., 2014). We did not find perceptible alterations in the tissue structure and morphology caused by the tissue volume expansion-contraction.

### 4.2. Computational quantification of sigmoid mucosal innervation and targeted cells in 3D images

Immunolabeling showed that NFs enter the colonic mucosa from the base and arborize in tortuous paths with their own distinct distribution patterns. PGP9.5, Calb and SP fibers spread evenly in the mucosa from the base to the top while hpChAT, NPY and TH fibers are visualized mostly in the lower area (Fig. 2A–G). The 2D imaging of these NFs with the mucosal sections crossing the different depths and in a suboptimal orientation would result in reducing visualization of overlapping structures, lacking representativeness and consistency of selected images for quantitation and comparison. To avoid the limitations associated with 2D images, we firstly created 3D images from Z-stack confocal images each with150–200 optical sections to provide enough depth for showing spatial views of each marker-labeled NFs, EGCs and MCs. Each 3D image with full-thickness mucosa was aligned with epithelia facing up vertically from the top to the base. Next, we adapted the Imaris 9.7–9.9 Surfaces Rendering Technology and developed a computerized approach for the measurement of volumes of surface-masked NFs and calculation of densities of NFs, EGCs and MCs in 3D images of the sigmoid mucosa biopsies. It is well established that MCs lie in proximity to NFs in most tissues including the human gut mucosa (Barbara et al., 2004, 2007; Li et al., 2022; Barreau et al., 2008) and release mediators including histamine, serotonin, proteases and neurotrophins that contribute to dysmotility and visceral pain by sensitizing enteric neurons and the terminals of sensory neurons in dorsal root ganglia (Barbara et al., 2007; Balemans et al., 2019; Cenac et al., 2010; Ostertag et al., 2017; Wouters et al., 2016). Mast cell activation and close proximity to nerve fibers in IBS patients correlated with worse abdominal pain severity (Dothel et al., 2015; Park et al., 2003). These are indicative of the importance of assessing the proximity of MCs to NFs as part of peripheral mechanisms relating visceral pain to IBS symptom severity. Currently, the method used to evaluate the proximity of MCs to NFs in human colonic biopsies mostly relies on the measurement of the distances between MCs to NFs imaged with electron microscopy under high magnification (x5300–9100) imaging of a very small field (total 90 μm^2^ per 10 fields) (Barbara et al., 2004) which restricted random sampling from whole mucosal biopsies. Importantly, the spatial configuration of MCs and NFs illustrated by 3D images and videos cannot be achieved on 2D electron micrographs. Imaris version 9.7 introduced the measurement of the shortest distance between spots and surfaces in 3D image analysis. This measurement enables the analysis of spatial relationships between spot objects and surfaces, allowing for filtering, segmentation, and quantitative analyses of object distributions and their proximity. The typical application of this method involves segmenting blood vessels as surface objects using Imaris’ surface rendering tools, then detecting individual microglia that exhibit dynamic and adaptable morphology, ranging from highly branched processes to elongated, rod-like, and amoeboid forms, as point objects through spot detection algorithms. This technique permits a detailed spatial analysis of how closely microglia are positioned relative to blood vessel surfaces (https://imaris.oxinst.com/versions/9–7). In our study, we developed an approach for the quantitative analysis of the proximity of MCs to nerve fibers in sigmoid mucosa biopsies by employing Imaris versions 9.7–9.9, using MCs as spot objects and nerve fibers as surface objects. Human colonic mucosa MCs are typically round or oval, ranging in size from 10 to 15 μm in diameter (Benditt and Lagunoff, 1964). Their shapes can vary slightly, sometimes appearing more elongated or spindle-like, as shown in Fig. 3C. To minimize errors when assessing the proximity of MCs (which exhibit varying morphologies) to nerve fibers, we measured both the long and short diameters of each mast cell, calculating an average diameter of 10.4 μm for this study. The shortest distances from the centers of each individual MC to the nerve fiber surfaces were automatically measured and plotted using Imaris versions 9.7–9.9. Thus, the shortest distance equal to or less than 5.2 μm was defined as contact with nerve fibers. These computerized measurements not only reduce biases due to observer/examiner judgment and overcome limitations of 2D images but also are much faster than measuring manually and facilitate the quantification of a large number of samples, increasing statistical accuracy.

The quantitative data from eight healthy adult participants including four of each sex showed distinct densities of 8 marker-labeled NFs which represent respectively the total, extrinsic sympathetic and parasympathetic, intrinsic and sensory innervation of the human sigmoid colonic mucosa. We found that the densities of intrinsic NFs labeled by Calb or VIP were significantly greater than those of extrinsic NFs labeled by TH and NPY or VAChT and SP (mostly extrinsic). To our knowledge, this is the first report in 3D structures showing a comprehensive and quantitative analysis of innervation of human colonic mucosa harvested from sigmoid mucosa biopsies. Data also showed that the proximity of MCs to Calb-labeled NFs is greater than that of MCs to SP-labeled NFs (p < 0.05). This may have a bearing with the greater density of Calb-than SP-labeled NFs and may also implicate more prominent crosstalk between MCs and intrinsic primary afferent nerves as Calb is a well-established immunohistochemical marker for intrinsic primary afferent neurons in the intestine (Brehmer et al., 2004; Weidmann et al., 2007). We did not find significant differences in all measurements between men and women. To the best of our knowledge, sex differences in the NFs, EGCs and MCs of human sigmoid mucosa is not known. It should be noted that the data obtained from the above quantitative analysis represent sampling from only a small group size used for the method development.

In conclusion, the novel method developed shows the capability of fast tissue clearing, which is compatible with immunolabeling and provides high-quality 3D imaging. In addition, we established the precise computational quantitation of NFs, related targeted cells and the proximity of MCs to the NFs in 3D structure of the human sigmoid mucosa biopsies. Computerized quantitation is much faster than measuring manually via grid-based stereology and histomorphometry and allows us to quantitate a larger number of samples with less bias. The parameters created in this approach also provide an efficient reference for applying the New AI Machine Learning Segmentation recently implemented into Imaris 10.1 for our quantitative analysis of large datasets. This new tool for the quantitative assessment of innervation and interactions of MCs and NFs can be applied to biopsies of patients with chronic visceral pain disorders, such as IBS, and has the potential to bring new insight for diagnostic and therapeutic purposes. Such approaches would also apply to mucosal biopsies from other regions of the GI tract, enabling not only the quantitation of innervation but also the assessment of vasculature, inflammation, and the tumor microenvironment.

## Supplementary Material

1

2

3

4

5

6

7

8

9

10

11

12

13

## Figures and Tables

**Table 1:** Immunofluorescent reagents.

	Name	Host	Source/ Catalog No.	Dilution
**Primary antibodies**	Protein gene product 9.5	Rabbit	Abcam/ ab108986	1:1000
	Tyrosine hydroxylase	Rabbit	Millipore/ AB152	1:1000
	Substance P	Rabbit	ImmunoStar/ 20064	1:1000
	Vasoactive intestinal peptide	Rabbit	CURE UCLA/ 7913	1:1000
	Vesicular acetylcholine transporter	Rabbit	Synaptic System/ 139 103	1:1000
	Human peripheral form of choline acetyltransferase	mouse	Dr. Bellier/ H3	1:2000
	Calbindin	Rabbit	Swant/ CB–38	1:1000
	Neuropeptide Y	Rabbit	CURE UCLA/ 8713	1:500
	S100β	Rabbit	Abcam/ ab52642	1:1000
	Mast Cell Tryptase, AA1	Mouse	Agilent Dako/ M7052	1:500
**Secondary antibodies**	Alexa 488-conjugated anti-mouse IgG	donkey	ThermoFisher Scientific/A–32766	1:400
	Alexa 555-conjugated anti-rabbit IgG	donkey	ThermoFisher Scientific/A–31572	1:400
**Normal serum**	Normal donkey serum	donkey	Jackson ImmunoResearch	1:10

## Data Availability

Data will be made available on request.

## Appendix A. Supporting information

Supplementary data associated with this article can be found in the online version at doi:10.1016/j.jneumeth.2025.110436.
