# Supplementary material for: Three-dimensional imaging and computational quantitation as a novel approach to assess nerve fibers, enteric glial cells, mast cells, and the proximity of mast cells to the nerve fibers in human sigmoid mucosal biopsies from healthy subjects
Source: J Neurosci Methods. Author manuscript; Available in PMC 2026 Jun 15. (PMC13267500; doi:10.1016/j.jneumeth.2025.110436)
Supplement: 1 [file NIHMS2173413-supplement-1.docx]

**SUPPLEMENTARY MATERIALS**

**CAPTIONS FOR SUPPLEMENTAL VIDEOS**

Video 1. A 360-degree visualization of the spatial relationship between mast cells (MCs) and nerve fibers (NFs) double-labeled with PGP9.5 and tryptase in a human sigmoid mucosa biopsy. This video was generated using Imaris 9.7-9.9 from a z-stack image comparable to the one used for Fig. 2A. The axes and rotations in the schematic are defined as follows:

X-axis: Represents the horizontal plane (left-right direction).

Y-axis: Represents the vertical plane (up-down direction).

Z-axis: Represents the depth plane (forward-backward direction).


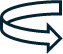
: 180-degree horizontal rotation


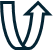
: 180-degree vertical rotation

Video 2. A 360-degree view of the spatial configuration of mast cells (MCs) and intrinsic primary afferent nerve fibers (NFs) double-labeled with substance P (SP) and tryptase in the human sigmoid mucosa biopsy. The video was created with Imaris 9.7-9.9 from a z-stack image similar to that used for Fig. 2B. The axes and rotations in the schematic are defined as follows:

X-axis: Represents the horizontal plane (left-right direction).

Y-axis: Represents the vertical plane (up-down direction).

Z-axis: Represents the depth plane (forward-backward direction).


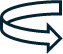
: 180-degree horizontal rotation


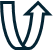
: 180-degree vertical rotation

Video 3. A 360-degree view of the spatial configuration of mast cells (MCs) and extrinsic primary afferent nerve fibers (NFs) double-labeled with calbindin (Calb) and tryptase in the human sigmoid mucosa biopsy. The video was created with Imaris 9.7-9.9 from a z-stack image similar to that used for Fig. 2C. The axes and rotations in the schematic are defined as follows:

X-axis: Represents the horizontal plane (left-right direction).

Y-axis: Represents the vertical plane (up-down direction).

Z-axis: Represents the depth plane (forward-backward direction).


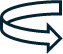
: 180-degree horizontal rotation


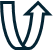
: 180-degree vertical rotation

Video 4. A 360-degree view of the intrinsic secretomotor nerve fibers labeled with vasoactive intestinal peptide (VIP) in the human sigmoid mucosa biopsy. The video was created with Imaris 9.7-9.9 from a z-stack image similar to that used for Fig. 2E. The axes and rotations in the schematic are defined as follows:

X-axis: Represents the horizontal plane (left-right direction).

Y-axis: Represents the vertical plane (up-down direction).

Z-axis: Represents the depth plane (forward-backward direction).


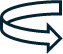
: 180-degree horizontal rotation


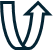
: 180-degree vertical rotation

Video 5. A 360-degree view of the extrinsic sympathetic nerve fibers labeled with neuropeptide (NPY) in the human sigmoid mucosa biopsy. The video was created with Imaris 9.7-9.9 from a z-stack image similar to that used for Fig. 2F. Video 6. A 360-degree view of the enteric glial cells labeled with S100β in the human sigmoid mucosa biopsy. The video was created with Imaris 9.7-9.9 from a z-stack image similar to that used for Fig. 2H. The axes and rotations in the schematic are defined as follows:

X-axis: Represents the horizontal plane (left-right direction).

Y-axis: Represents the vertical plane (up-down direction).

Z-axis: Represents the depth plane (forward-backward direction).


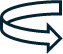
: 180-degree horizontal rotation


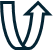
: 180-degree vertical rotation

Video 6. A 360-degree view of the enteric glial cells labeled with S100β in the human sigmoid mucosa biopsy. The video was created with Imaris 9.7-9.9 from a z-stack image similar to that used for Fig. 2H. The axes and rotations in the schematic are defined as follows:

X-axis: Represents the horizontal plane (left-right direction).

Y-axis: Represents the vertical plane (up-down direction).

Z-axis: Represents the depth plane (forward-backward direction).


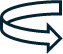
: 180-degree horizontal rotation


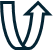
: 180-degree vertical rotation

| Marker antibodies^a^ | Healthy subjects n=8^b^ | Men n=4^c^ | Women n=4^d^ |
| --- | --- | --- | --- |
| PGP9.5 | 2.44±0.23 | 2.42±0.34 | 2.46±0.37 |
| Calb | 1.88±0.19 | 2.11±0.16 | 1.62±0.31 |
| VIP | 1.50±0.0.25 | 1.75±0.45 | 1.25±0.21 |
| hpChAT | 0.40±0.05 | 0.44±0.10 | 0.36±0.05 |
| TH | 0.38±0.03^***, ###^ | 0.38±0.03 | 0.39±0.07 |
| VAChT | 0.24±0.05^***, ###^ | 0.25±0.09 | 0.22±0.06 |
| NPY | 0.40±0.05^***, ###^ | 0.38±0.06 | 0.42±0.07 |
| SP | 0.90±0.16^***^ | 0.89±0.25 | 0.91±0.23 |
| S100 | 2.02±0.18 | 1.93±0.33 | 2.11±0.22 |
| Tryptase | 1.61E-05±1.23E-06 | 1.73E-05±2.01E-06 | 1.50E-05±1.50E-06 |
| PGP9.5-MC | 60.45±4.10 | 57.32±4.61 | 63.58±7.12 |
| SP-MC | 44.10±5.35^+^ | 37.44±3.93 | 50.77±9.40 |
| Calb-MC | 65.25±4.97 | 65.57±7.80 | 64.94±7.37 |

**Supplemental Table 1**. Quantitative analysis of nerve fibers, enteric glial cells, mast cells and proximity of mast cells to nerve fibers in the sigmoid mucosal biopsies from adult health subjects (mean±SEM).

Quantitative analysis of densities of nerve fibers (NFs), enteric glial cells (EGCs), mast cells (MCs) and proximity of MCs to NFs in 3D images of human sigmoid colonic mucosa biopsies using the computerized approach developed in this study. ^a^The NFs were subclassified by immunolabeling with specific marker antibodies against protein gene product 9.5 (PGP9.5) for pan-NFs, calbindin (Calb) for intrinsic primary afferent NFs, vasoactive intestinal peptide (VIP) for intrinsic secretomotor NFs, human peripheral choline acetyltransferase(hpChAT) for intrinsic cholinergic NFs, vesicular acetylcholine transporter (VAChT) for cholinergic NFs with extrinsic and intrinsic origin, neuropeptide (NPY) and hydroxylase (TH) for sympathetic NFs and substance (SP) for extrinsic primary NFs. EGCs and MCs were labeled with S100β and Tryptase, respectively. ^b^The densities of NFs and EGCs (v/v, %), MCs (MCs/µm^3^) and the proximity of MCs to PGP9.5, Calb and SP-labeled NFs (MC-PGP9.5, MC-Calb, MC-SP) (% of MCs with contact to NFs in the total MCs) were calculated from 5-6 3D images labeled with each marker per subject and averaged from 12 adult health subjects including 6 of each sex. Statistical analysis of the difference between two groups was performed by two-tailed student's t-test. Multiple group comparisons were performed with a one-way analysis of variance (ANOVA) followed by Tukey’s post hoc test. Data were expressed as mean ± SEM. *** p<0.001 vs Calb; # p<0.05, ### p<0.001 vs VIP; + p<0.05 vs. Calb-MC. ^c, d^ Data were averaged from six of each sex. No significance was detected between sex.
