# Supplementary figures and images for: Three-dimensional imaging and computational quantitation as a novel approach to assess nerve fibers, enteric glial cells, mast cells, and the proximity of mast cells to the nerve fibers in human sigmoid mucosal biopsies from healthy subjects
Source: J Neurosci Methods. Author manuscript; Available in PMC 2026 Jun 15. (PMC13267500; doi:10.1016/j.jneumeth.2025.110436)

## Slide 1
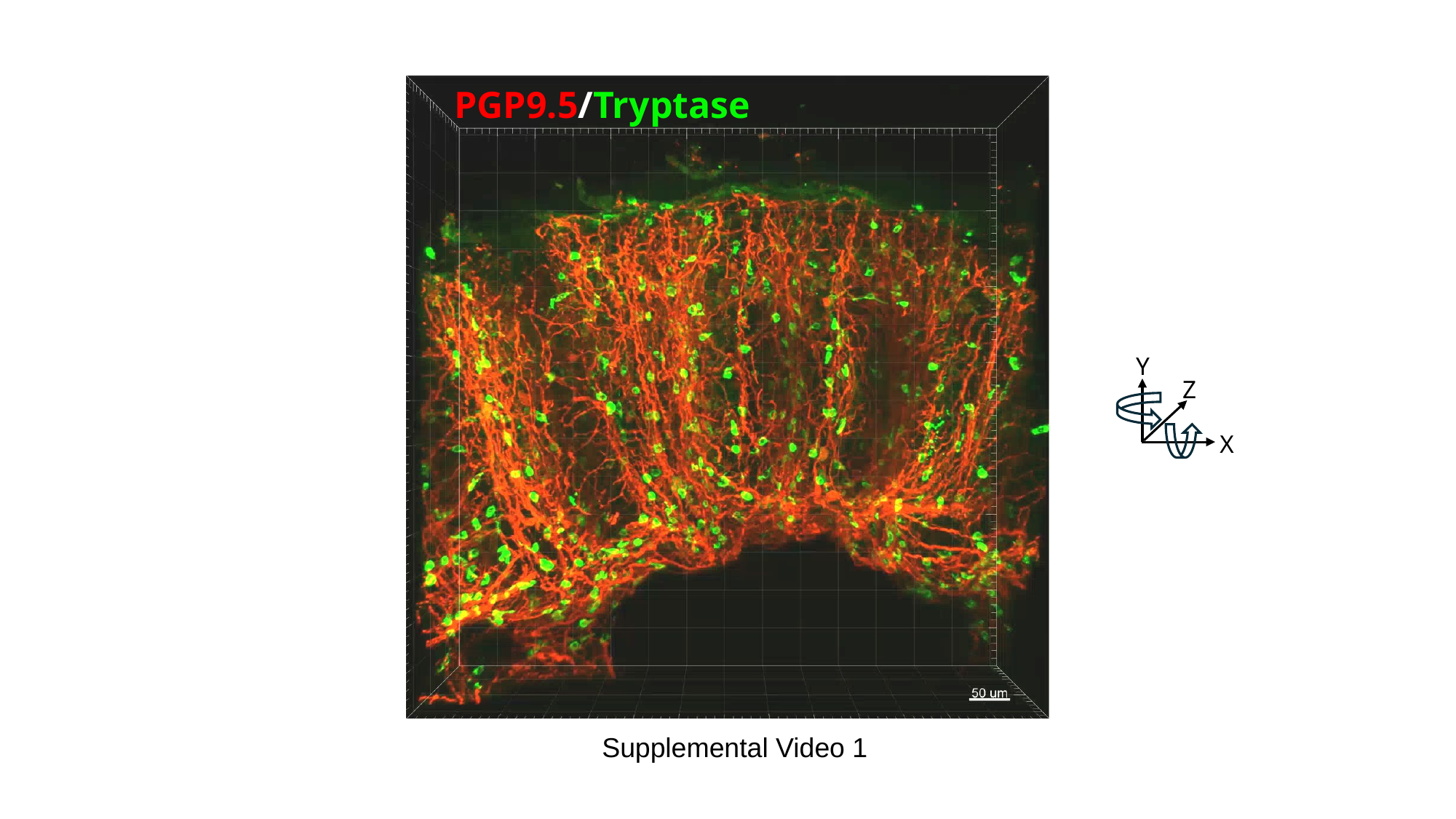

PGP9.5/Tryptase
Y
Z
X
Supplemental Video 1

Supplement: 6 [file NIHMS2173413-supplement-6.pptx]

## Slide 1
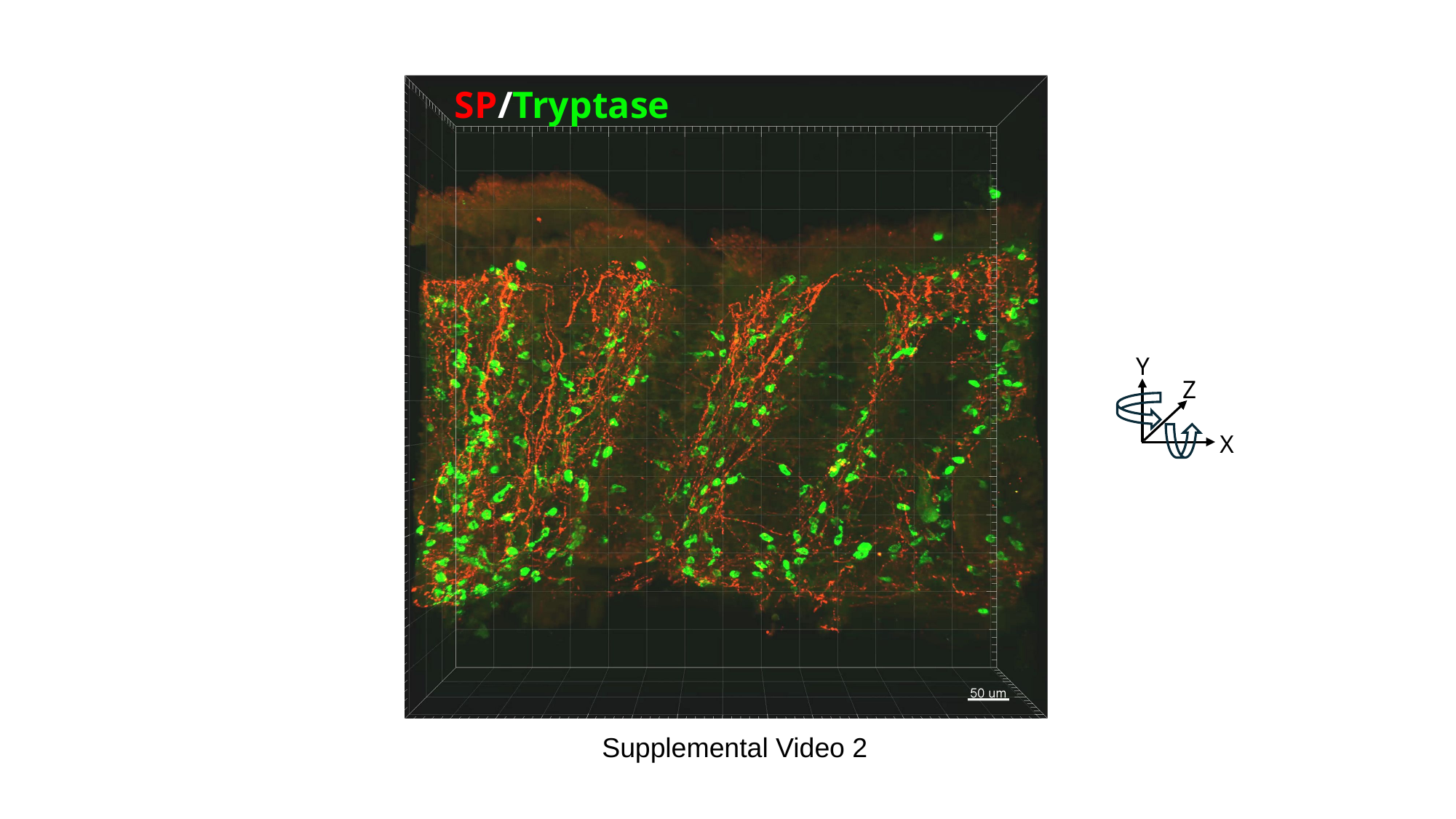

SP/Tryptase
Y
Z
X
Supplemental Video 2

Supplement: 7 [file NIHMS2173413-supplement-7.pptx]

## Slide 1
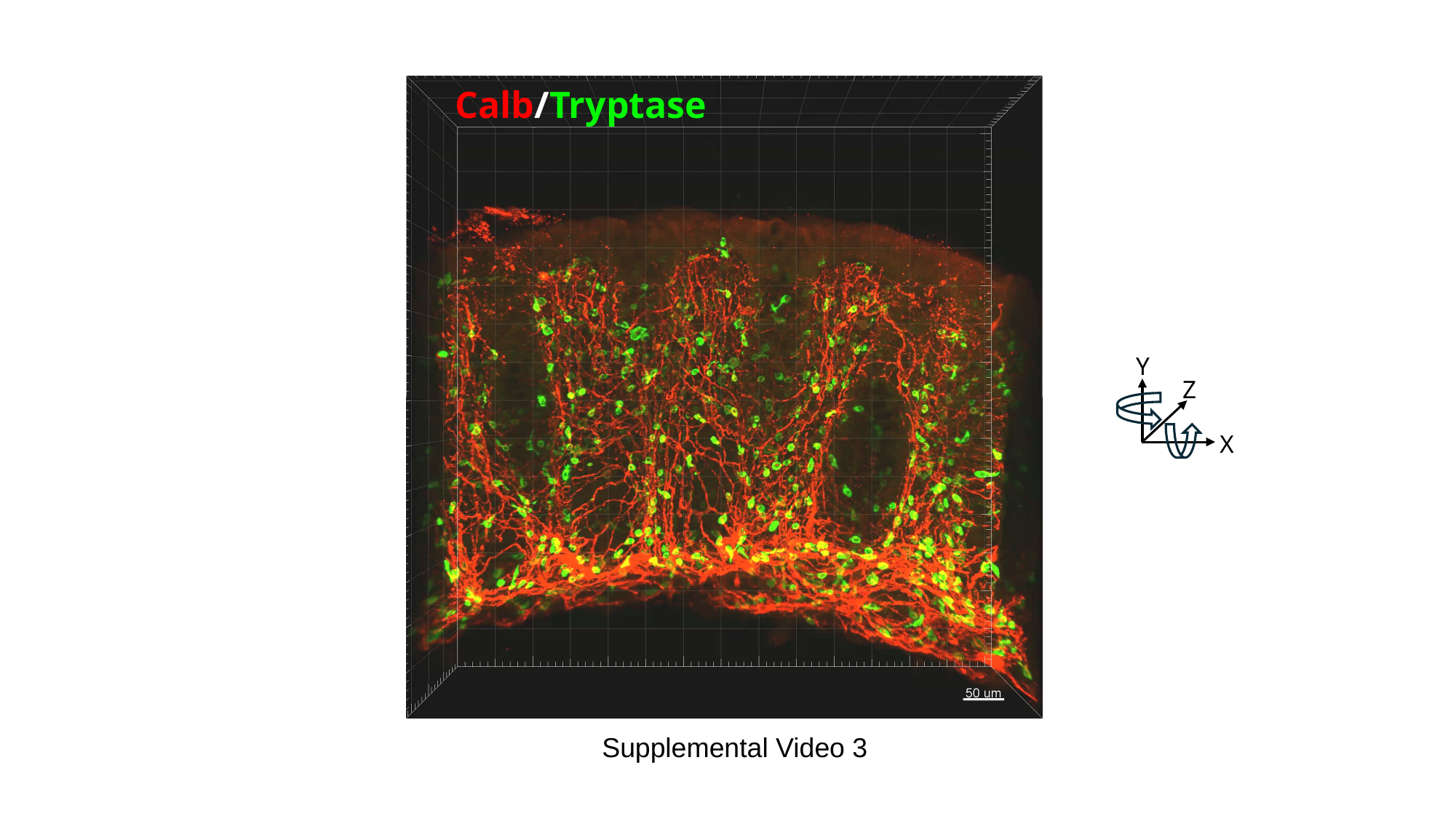

Calb/Tryptase
Y
Z
X
Supplemental Video 3

Supplement: 8 [file NIHMS2173413-supplement-8.pptx]

## Slide 1
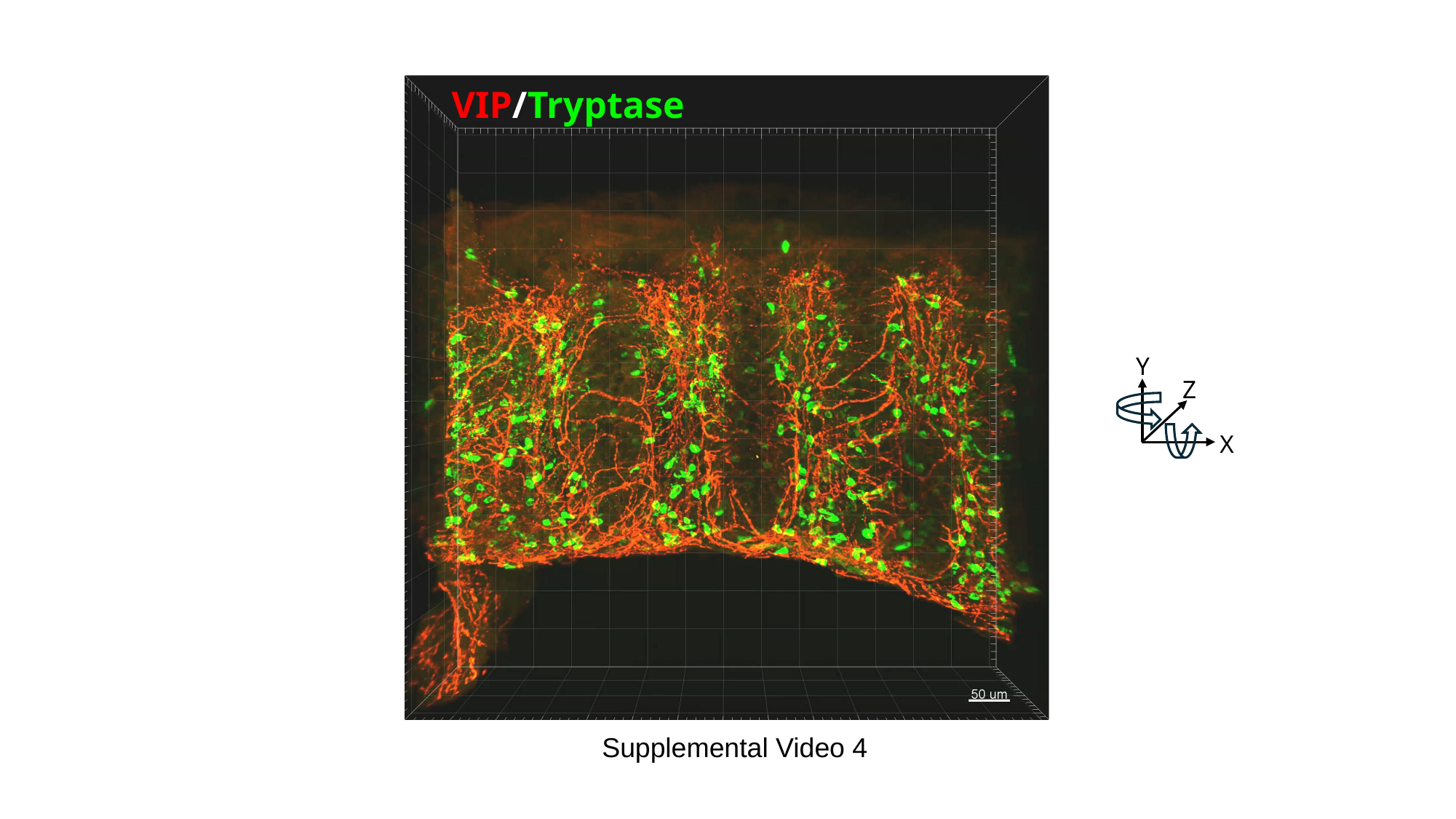

VIP/Tryptase
Y
Z
X
Supplemental Video 4

Supplement: 9 [file NIHMS2173413-supplement-9.pptx]

## Slide 1
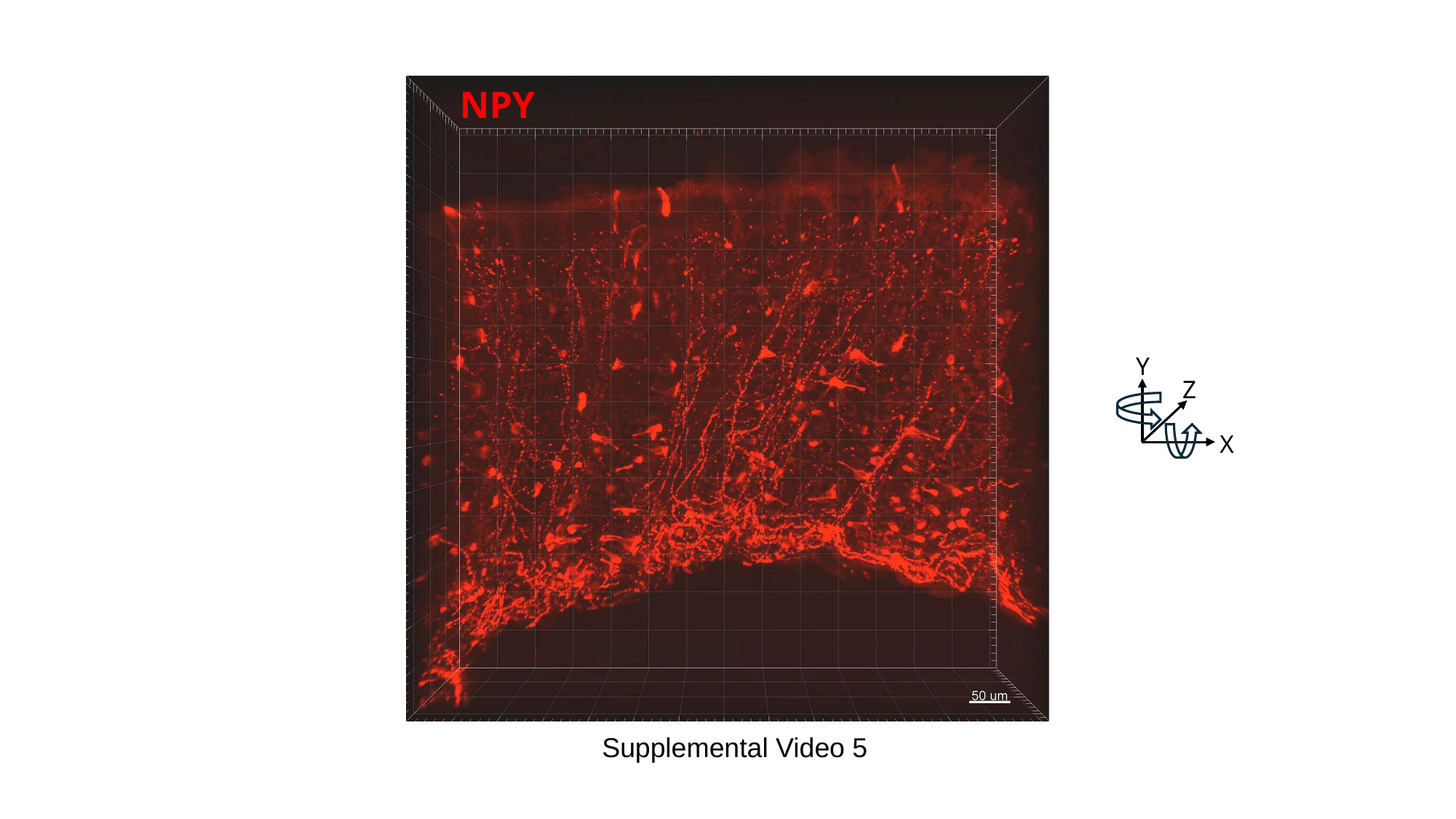

NPY
Y
Z
X
Supplemental Video 5

Supplement: 10 [file NIHMS2173413-supplement-10.pptx]

## Slide 1
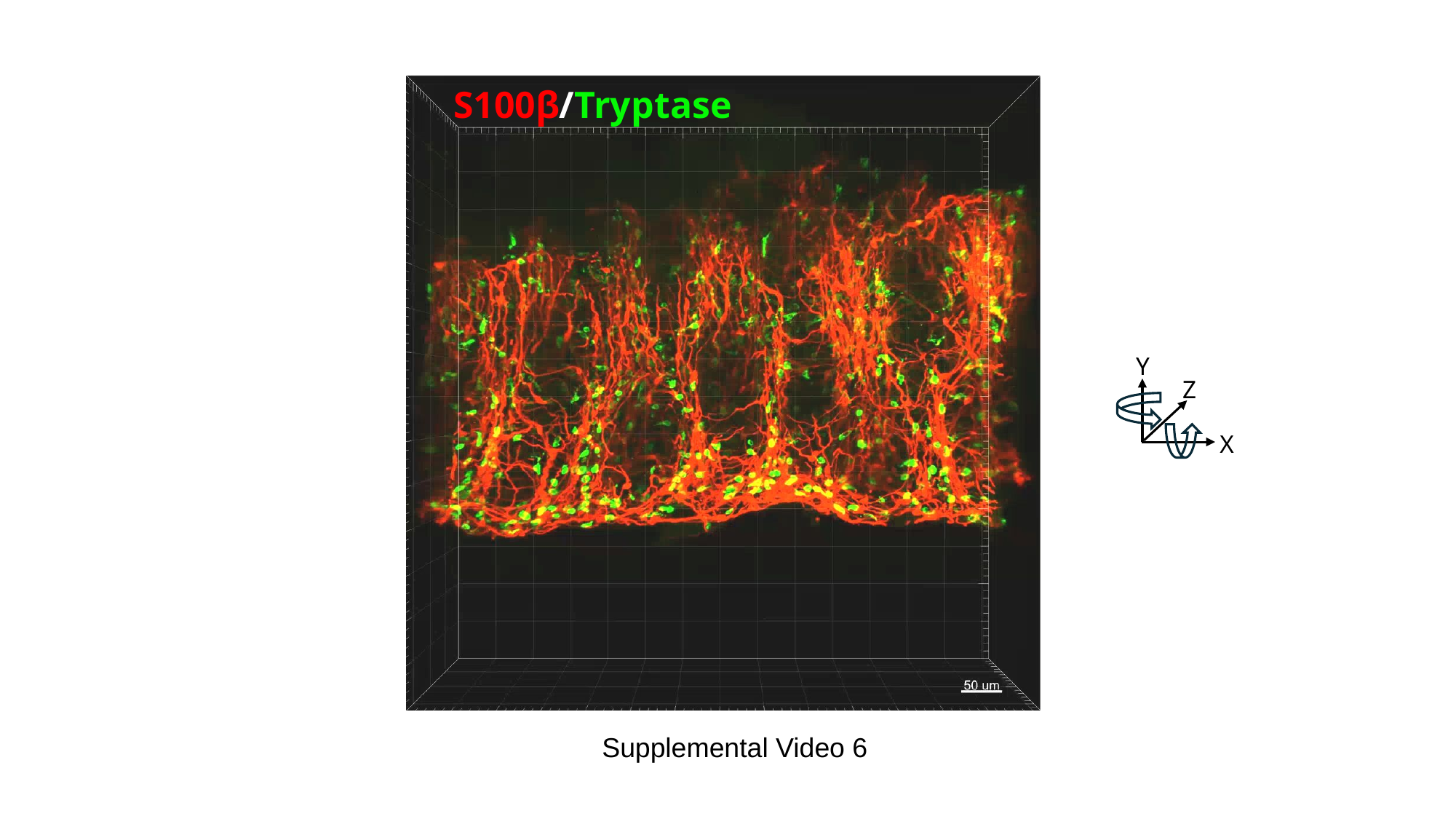

S100β/Tryptase
Y
Z
X
Supplemental Video 6

Supplement: 11 [file NIHMS2173413-supplement-11.pptx]
